# Some Points on Cleansing the Ear

**Published:** 1881-05

**Authors:** 


### SOME POINTS ON CLEANSING THE EAR.

Dr. W. H. Bennett, of Brooklyn, writes: "Books on aural diseases, and ear specialists generally, advocate for cleansing the auditory canal the use of a syringe, having a short, blunt, or swollen nozzle, and advise further, simply that the canal should be thoroughly straightened while using the instrument. But experience has taught me that this is not the proper syringe, and that a somewhat different method should be pursued. We want free vent for the outflow, or return current, of water (except in the comparatively few cases where it is desired to force the fluid through a perforated drum-head into the parts beyond), in order that it may carry out the foreign body, wax, or pus ; and then we should be able to *see* what we are about, and to direct the stream with exactness and nicety above, below, or on either side of the substance to be removed, if it be impacted in the canal, and so cause the fluid to accumulate behind it without forcing it farther in. To accomplish these ends we must use the head-mirror, the speculum, and a syringe with a long, slender nozzle. I use the ordinary uterine syringe. By means of these instruments, one can syringe an ear neatly, intelligently, and effectually, and there is no more danger to the patients, nor as much, as by the old method. To my mind it is as unphilosophical to syringe an ear in the dark as it would be to probe the auditory canal blindfolded ; and should the army of ear-syringers kick against the method here recommended, I can only assure them that when any one of their number has tried it he will adopt it. The stream of water should not be thrown with quite so much force as is generally employed, for it impinges more directly upon the drum-head or walls of the middle ear, and of course the smaller the stream, with the same amount of power, the swifter the current and the more violent its action. The speculum should be as large as it is practicable to use. During the syringing it will frequently fill with water and obstruct the view, but this may be sucked out and the parts again exposed to the eye. If it be a foreign body or lump of wax we are endeavoring to remove, as it makes its way out the speculum may be gradually withdrawn until the substance reaches a point where it may be seized by the forceps, or admit of the introduction of a probe behind it. It is understood of course, that the syringe, and method of using it, here mentioned, is meant to be intrusted only to professional men, as it would be a dangerous instrument in the hands of a layman. A few words regarding the drying out of the canal after syringing, which ought always to be done, except in acute inflammations. For mopping up the residual fluid a camel's-hair brush of good size, previously moistened and squeezed out, answers the purpose admirably. It can be sufficiently dried by pinching it between the folds of a towel, and may be repeatedly used. Afterward, for removing every trace of moisture, ordinary blotting-paper, cut into narrow strips, a line or two wide, is the most convenient, and by far least dangerous or irritating material I have found.—*The Medical Record.*

